# Lattice Boltzmann modeling to explain volcano acoustic source

**DOI:** 10.1038/s41598-018-27387-0

**Published:** 2018-06-22

**Authors:** Federico Brogi, Maurizio Ripepe, Costanza Bonadonna

**Affiliations:** 1 Department of Earth Sciences, University of Geneva, Geneva, Switzerland; 2 Dipartimento di Scienze della Terra, Università di Firenze, Firenze, Italy; 3 Istituto Nazionale di Oceanografia e di Geofisica Sperimentale, Sgonico, Italy; 4 Istituto Nazionale di Geofisica e Vulcanologia, Sezione di Pisa, Italy

## Abstract

Acoustic pressure is largely used to monitor explosive activity at volcanoes and has become one of the most promising technique to monitor volcanoes also at large scale. However, no clear relation between the fluid dynamics of explosive eruptions and the associated acoustic signals has yet been defined. Linear acoustic has been applied to derive source parameters in the case of strong explosive eruptions which are well-known to be driven by large overpressure of the magmatic fluids. Asymmetric acoustic waveforms are generally considered as the evidence for supersonic explosive dynamics also for small explosive regimes. We have used Lattice-Boltzmann modeling of the eruptive fluid dynamics to analyse the acoustic wavefield produced by different flow regimes. We demonstrate that acoustic waveform well reproduces the flow dynamics of a subsonic fluid injection related to discrete explosive events. Different volumetric flow rate, at low-Mach regimes, can explain both the observed symmetric and asymmetric waveform. Hence, asymmetric waveforms are not necessarily related to the shock/supersonic fluid dynamics of the source. As a result, we highlight an ambiguity in the general interpretation of volcano acoustic signals for the retrieval of key eruption source parameters, necessary for a reliable volcanic hazard assessment.

## Introduction

The classical acoustic theory directly relates the time evolution of the volumetric flow rate with the waveform of the first acoustic perturbation. The simple source model has been used extensively not only to explain infrasonic signals generated by discrete volcanic explosions but also to retrieve key eruption source parameters (e.g. mass eruption rate)^1–8^. However, several assumptions and simplifications have to be made in order to derive the source model from the general non linear equations (Navier-Stokes equations) describing the dynamics of a fluid^9–11^. In particular, all the physical quantities involved in the motion such as air density, velocity of the air parcel (*u*), atmospheric pressure and air temperature are considered to undergo small fluctuations over a reference value. This assumption is valid for most acoustic phenomena involving pressure fluctuations orders of magnitude smaller than the atmospheric pressure. Furthermore, the local air velocity induced by the volumetric flux should be much smaller than the sound speed (*c*_*s*_), equivalent to low acoustic Mach number $(\mathrm{Ma}=u/{c}_{s}\ll 1)$ reducing the application of this theory to the sub-sonic explosive dynamics. These conditions are typically violated in the presence of strong pressure transients, like shock waves that propagates at supersonic speeds. Non-linear propagation effects start to be important when the excess pressure (*δp*) induces a significant temperature change, which considerably affects the sound speed. Large explosions, such as chemical and nuclear explosions, produce an initial symmetric pressure pulse with very large *δp* values. For these cases, since high-pressure portions of the pulse travel at higher speed than the low-pressure portions, the wave front steepens progressively as it propagates forming a vertical shock discontinuity. However, when traveling away from the source, shock waves lose this asymmetry and the waveform take the typical symmetric N-wave shape. The critical distance for this change to happen depends on the excess pressure at the source^12^. During large Vulcanian and Plinian eruptions, discrete explosive pulses release large quantities of gas in a very short time, and therefore, generate pressure pulses characterized by large *δp* values. As an example, explosive events at the Mount St Helens in 1980 produced transients with 5 kPa of peak-to-peak pressure at 54 km^13^. Infrasonic signals associated with these big events have been recorded mostly at distances far enough from the source (>4 km) to be characterized by N shaped waveforms^14^. Infrasound recordings for much smaller explosive events associated with Strombolian activity are instead available for shorter distances from the source (<1 km). Although symmetric waveforms commonly characterize these signals, asymmetric transients have also been recorded during Strombolian explosions at Erebus (Antartica)^6^, Yasur (Vanuatu)^15^, Karymsky (Kamchatka)^16^, Tungurahua (Ecuador)^17^, Nabro (Eritrea)^18^ and Stromboli (Italy)^19^ volcanoes. Infrasonic signals characterized by an asymmetric waveform have been explained as produced by the diffraction of crater rim^20^, whereas in some cases the asymmetric waveform is shown to be remarkably similar to the Friedlander waveform^15^. In this latter case, the striking similarity between waveforms may represent the evidence of a blast wave generated by the supersonic dynamics of small but still violent Strombolian explosions^15^ and it is confirmed by the slightly (345–437 ms^−1^) supersonic propagation speed of the measured acoustic front. This means that in absence of complementary measurements on the explosive dynamics such as in the case of Yasur^15^, it is very difficult to discriminate between linear and non-linear source dynamics from the acoustic waveform and further investigations are needed.

Fluid dynamics, including wave related phenomena, can be either described by the Navier-Stokes equations (NS) or by a discrete model of interacting particles, such as the Lattice Boltzmann method (LBM)^21^. While NS solvers tracks the evolution of macroscopic observables (e.g. density, velocity), the LBM deals with mesoscopic quantities (particle density distribution functions), relying on the solution of a discrete form of the Boltzmann Equation. The main advantage of LBM over the NS solvers is the absence of non-linearity in the convection term and the need to solve the Poisson equation for obtaining pressure. In addition, the LBM has been proven to have the low dissipative properties needed for modeling the weak acoustic pressure fluctuations. Such characteristics make the LBM, easy to code, very good for parallelization and efficient for solving complex fluid flows including the generation and propagation of acoustic waves.

In this work, we review the conceptual model of the subsonic injection of volcanic gas as a source of infrasonic transients by using a numerical model based on the Lattice-Boltzmann method. We show how the recorded acoustic waveforms can be explained as the result of the velocity profile driven by the eruptive flow. We show that the blast/shock wave dynamics or the diffraction around the source is not a necessary requirement for explaining the asymmetric waveform of acoustic waves sometimes observed at explosive volcanoes during moderate Strombolian explosions.

## Results

**Figure 1:**
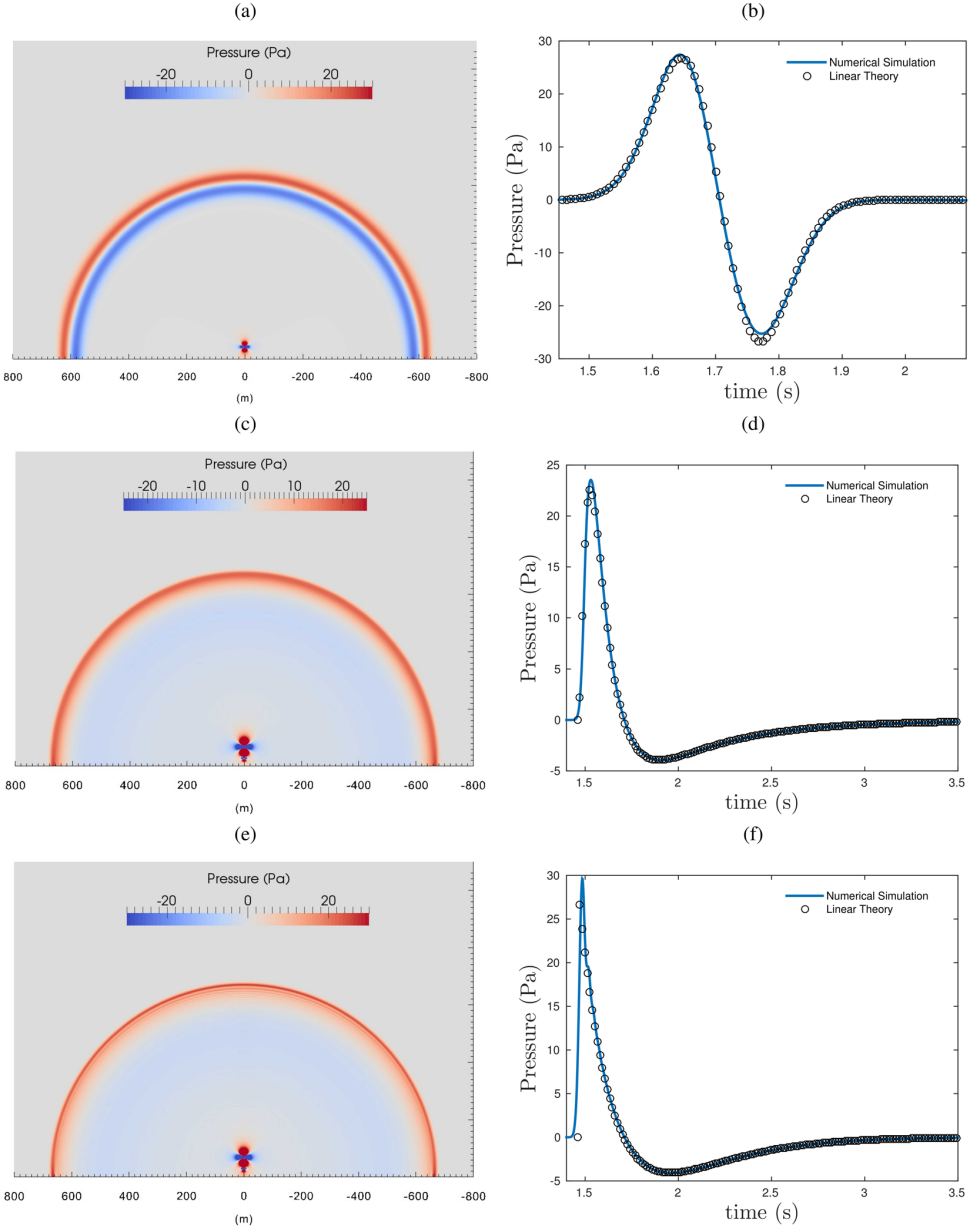
3D Numerical simulation using our LBM scheme for an injection of a fluid in an ambient fluid at rest for symmetric (Gaussian: **a,b**) and asymmetric Log-Normal (**c,d**) and Friedlander-derived (**e,f**) SRT. The computational domain is 1 × 1 × 1 km, the jet inlet (D = 10 m) is placed at the bottom boundary in the center with a diameter of 10 m. The red region near the center of the bottom axis is the hydrodynamic pressure field generated by the starting jet (vortex). Slices of the synthetic wave fields (**a,c,e**) and synthetic infrasonic signals at a radial distance of 500 m from the vent are shown (**b,d,f**). In the slices (**a,c,e**) the vertical scale is the same as the horizontal one.

The motion of the fluid produced by a volcanic explosion may be strongly directed upward and is in general affected by non-linear phenomena related for instance to compressibility and development of flow instability. Thus, the question arises if the acoustic transient generated by the volcanic gas and particulate jet may deviate from the one predicted by the linear theory. Using a numerical fluid solver based on the Lattice Boltzmann method, which is not affected by the assumptions of the acoustic linear theory, we can explore the origin of the acoustic transient generated by subsonic injections. 3D LBM modeling of subsonic fluid injections in homogeneous ambient fluid at rest are reported in Fig. 1. In order to reproduce a reliable magmatic fluid injection, a choice for the evolution of the volumetric flow rate, the source time function (SRT), need to be made. Following the linear theory of sound^22^, the most used volumetric flow rate in volcano acoustics is generally symmetric (e.g. ref.^20^) and has a Gaussian shape:

1 $${\dot{V}}_{g}(t)={e}^{-\frac{{(t/\tau -{\mu }_{g})}^{2}}{2{\sigma }_{g}^{2}}}$$

where *μ*_*g*_ and *σ*_*g*_ are the parameters for the Gaussian function and *τ* the rise time, the time needed for the SRT to reach is maximum value. While the vent diameter (D = 10 m), the peak exit velocity (100 m/s) and the rise time (0.25 s) are fixed, a time variable vertical velocity (hyperbolic radial profile) boundary condition (zero radial velocity) allows the gas flow rate to vary during the computation following an “a-prior” defined SRT. Let us note, that the rise time is chosen to have a fluid injection which can be considered as a compact source. Results of the modeling with a Gaussian SRT are visualized by snapshots of the wave field (Fig. 1a). For radial distances $r > \lambda$ from the vent, the amplitude of the first acoustic onset generated by the jet is the same in all the directions around the conduit and the wave length is roughly 10 times larger than the jet diameter. Taking the latter as a reference length, we conclude that at this frequency the extended fluid flow can also be approximated to the compact acoustic source, fully compatible within the classical assumption of the linear acoustic theory (Fig. 1b). Besides, at far field distances, the source could be considered a monopole.

### Acoustics of a subsonic directional fluid/mass injection

**Figure 2:**
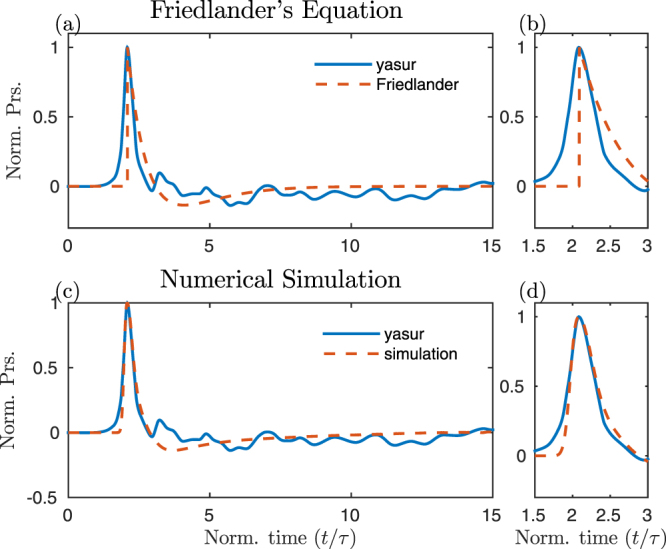
Comparison between the pressure waveform for an explosion infrasonic signal recorded at Yasur[15] and: (**a**) the Friedlander’s waveform describing blast waves, (**c**) the computed waveform from a 3D numerical simulation for an injection of fluid with a Log-normal SRT (aligned on the peak). The zoom of the compressive phase of the signals are also shown (**b,d**).

A rapid variation in the volumetric flow rate represent one of the most efficient mechanisms for fluid flow to produce pressure waves. It is well known, that volcanic explosive eruptions result in a jet flow characterized by a rapid change in the flow rates^23^. Based on laboratory experiments where a volume of pressurized fluid (water) is suddenly released in a lower pressure environment, Chojnicki *et al*.^24^ propose the SRT for discrete explosive events to be well described by a Gaussian shape. This seems consistent with SRT used to model the source of volcano acoustic wavefield by Finite Domain Time Difference^20,25^ (FDTD). We thus assumed that our magmatic fluid flow is well represented by a Gaussian velocity profile with mean *μ*_*g*_ = 1 and standard deviation *σ*_*g*_ = 0.25. The synthetic signal for a distance of *r* > 2*λ* from the vent, has positive and negative phases of equal amplitude and duration, and is completely symmetric as expected from linear theory. In addition, the pressure transient propagates at sonic speed (Supplementary Fig. S1). Although further investigations are needed to better understand the physical constraints to assume a certain profile for the SRT, we now simply explore how the change in the shape of the SRT can reflect changes in the recorded infrasonic signals for subsonic fluid injections. In particular, we consider the case where the SRT is not represented by a symmetric Gaussian function but by an asymmetric Log-normal function:

2 $${\dot{V}}_{l}(t)={e}^{-\frac{{(\mathrm{ln}(t/\tau )-{\mu }_{l})}^{2}}{2{\sigma }_{l}^{2}}}$$

with the parameters *μ*_*l*_ = 0 and *σ*_*l*_ = 1, for an asymmetric Log-normal function. The acoustic wave does not show any significant directivity (Fig. 1c), the source is compact, and within the classical theory could be considered as a monopole. However, the waveform of the synthetic signal is remarkably different from the one generated by a symmetric SRT (Fig. 1d). The waveform is characterized by a sharp onset, short compressive phase and a smaller and longer rarefaction phase. Therefore, as expected from the linear theory, the degree of symmetry of the waveform simply reflects the degree of symmetry of the SRT. In addition, the waveform generated by a log-Normal SRT is remarkably similar to the one that typically characterizes blast waves, when measured not too far from the explosive source. Pressure waves generated by supersonic explosion, are in fact, characterized by sharp vertical onset followed by a much longer and smaller rarefaction phase^26^. The waveform of blast waves is known to be independent of the peak source overpressure (self similarity) and is well described by the Friedlander’s equation:

3 $$\delta p=\delta {p}_{s}{e}^{-t/\tau }(1-\frac{t}{\tau })$$

where *δp* is the peak source overpressure and *τ* the relaxation time, that is the duration of the positive part of the signal. As for the waveform generated by a Log-normal SRT, the asymmetry is a striking feature of the Friedlander’s function (Figs 2a and 1e,f for the LBM modeling), which can be also represented by a log-normal SRT. This result indicates that asymmetric acoustic waveform can be also explained in terms of the velocity profile of the fluid flow and not necessarily due to diffraction of the crater rim^20^ and/or supersonic dynamics^15^.

### Comparing fluid/mass flow injection to the simple source of the linear theory

**Figure 3:**
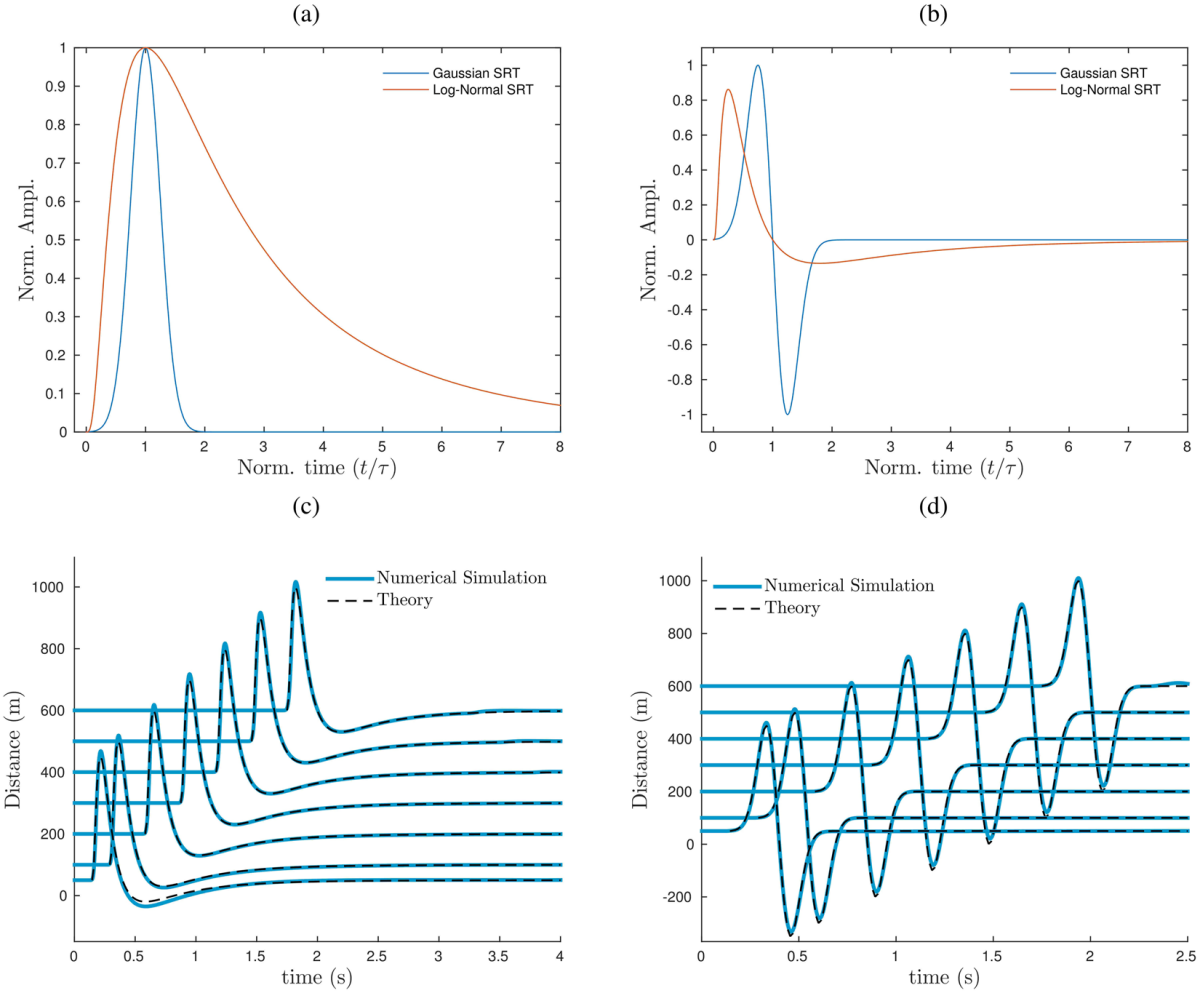
(**a,b**) Modeling results, according to the linear theory of sound, for symmetric and asymmetric acoustic transients from an injection of fluid in a three dimensional uniform half-space. (**c**,**d**) Comparison between the signals computed with the LBM and the one predicted by the linear theory (eqs 5, 6) at different radial distances from the source for: (**c**) the symmetric Gaussian SRT (*D* = 10 m, *U*_*max*_ = 100 m/s, *μ*_*g*_ = 1, *σ*_*g*_ = 0.25, *τ* = 0.25) (**d**) the asymmetric Log-Normal SRT (*D* = 10 m, *U*_*max*_ = 100 m/s, *μ*_*l*_ = 0, *σ*_*l*_ = 1, *τ* = 0.25). The amplitude of the computed signals is normalized based on the peak amplitude of the theoretical ones.

The results of the Lattice Boltzmann simulation have been compared with the more classical acoustic theory, which provides a simple way to relate the time evolution of a volumetric flow rate $\dot{V}(t)$, or SRT, with the waveform of the acoustic perturbation. Considering a simple source:

4 $$\ddot{V}(t)=\frac{2\pi r}{\rho }\delta p(t+r/{c}_{s},r),$$

where is *c*_*s*_ the sound speed, *ρ* the fluid density, *r* the radial distance from the source and *δp* = *p* − *p*_0_ the acoustic pressure (*p*_0_ the ambient pressure). In linear theory, the typical dimension (*d*) of the fluid region involved in the sound generation should be small with respect to the radiated wave length (*λ*, $d\ll \lambda$ for source compactness), and the source-receiver distance (*r*) should be much larger than the radiated wave length ($r\gg \lambda$, far field). In addition, the motion of the fluid should not have any preferred direction, and the expected waveform and amplitude would not depend on the azimuth but only on the source-receiver distance. Finally, non linear source processes are not explicitly included in eq. 4 and the waveform of the acoustic signal is determined by the derivative of the volumetric flow rate, the SRT. According to the simple source model (eq. 4) for a Gaussian SRT (Fig. 3a), the generated acoustic transient is described by:

5 $$\delta p(t)=-\,\frac{\rho }{2\pi r}\frac{t/\tau -{\mu }_{g}}{\tau {\sigma }_{g}^{2}}{\dot{V}}_{g}(t)$$

which represents an acoustic pulse, where both the amplitude and the duration of the positive and negative phases are equal (Fig. 3b). Let us note, that within this framework a finite fluid injection in a three dimensional space always implies a rarefaction phase to be present in the signal and that in the above equation the propagation time-lag (*r*/*c*_*s*_) is not considered since does not affect the waveform but only the arrival time. No propagation effects on the waveform are taken into account, as the wave is expected to propagate into an infinite or semi-infinite space.

We can then compare the signals predicted by the linear theory with the ones predicted by the numerical simulation using a Gaussian SRT (Fig. 3c). In order to highlight even small differences, the numerical signals have been normalized with respect to the maximum value of the theoretical ones. The latter have been calculated with eq. 5 and properly corrected for distance using the propagation time lag (*r*/*c*_*s*_). For this scenario, the synthetic wave field is in excellent agreement with the theoretical one, even for small distances from the source ($r > \lambda$). In addition, the synthetic transients travel at sonic speeds (Supplementary Fig. S1) and therefore the arrival times at different radial distances are consistent with the theory. Finally, we also consider the linear theory for the simulation with an asymmetric SRT. According to the simple source model (eq. 4) for a Log-Normal SRT (Fig. 3a), the generated acoustic transient is:

6 $$\delta p(t)=-\,\frac{\rho }{2\pi r}\frac{\mathrm{ln}(t/\tau )-{\mu }_{l}}{t{\sigma }_{l}^{2}}{\dot{V}}_{l}(t)$$

which describes an asymmetric waveform with a sharp onset, short compressive phase and a smaller and longer rarefaction phase (Fig. 3b). As in the previous case, except for a weak spurious directivity, the synthetic wavefield agree very well with the one predicted by the linear theory for a monopole source (Fig. 3b). Consistently with the predictions of the linear theory and the numerical results, the degree of symmetry of the SRT is reflected by the degree of symmetry of the waveform. In addition, the asymmetric waveform, either computed with linear theory or with the LBM, results to be remarkably similar to the Fridlander’s waveform. Therefore, it is interesting to consider a numerical simulation for a subsonic log-normal SRT fluid injection with a rise time consistent with one inferred by the thermal camera analysis of a strombolian explosion at Yasur volcano, for which supersonic dynamics has been inferred^15^. According to ref.^15^, the jet diameter in our LBM simulation is fixed at 15 m and the jet peak velocity of the ash and lapilli fluid at 190 m/s. The modeling shows an asymmetric synthetic signal with positive peak of 0.2 s in agreement with the infrasonic signal recorded at Yasur (Fig. 2). The amplitude of the computed transient is very sensitive to the value chosen for the vent diameter. For instance, following the linear theory and considering diameter values within the estimated range from thermal data, a vent diameter of 15 m produces a transient of ∼90 Pa at 700 m, almost double the amplitude of the one generated for a diameter of 11 m (∼50 Pa at 700 m) that is in agreement with the recorded signal at Yasur. Discrepancies in the negative part of the signal may be due to the presence of propagation effects in the recorded signals^15^ that are not taken into account in the simulation. This demonstrates the complexity of determining whether the source process is driven by subsonic or supersonic dynamics from the symmetry of the infrasonic waveform, but it also calls for careful consideration when the linear theory is used to invert for source parameters. This is more evident when the following function:

7 $$\delta p(t)=\frac{t}{\tau }{e}^{(1-\frac{t}{\tau })}$$

is considered. This represents, in fact, the SRT of the Fridlander’s waveform (eq. 3) where *τ* is the relaxation time representing the time at which the pressure crosses the zero^15^. The LBM modeling for subsonic fluid injections assuming *τ* = 0.25 s generates an acoustic signal with the sharp offset typical of supersonic dynamics. Besides, the result of the LBM is coherent with the linear theory and shows a pressure discontinuity (Fig. 1e,f) which reveals a paradox in our understanding of the acoustic signal associated with the explosive dynamics.

Finally, we remark that for Ma higher than 0.5 the wavefield presents a directivity (Supplementary Fig. S2) which instead is nearly absent at Ma (0.3). In particular, the generated acoustic field for the explosion at Yasur volcano (Ma = 0.55) is characterized by higher amplitudes in the first compression phase at small angles from the vertical axis (jet axis). Although the LBM model used in this work may be not fully accurate at higher Ma, we suggest that compressibility effects may be the cause of the observed directivity and that at even higher Ma the radiated acoustic field may be inconsistent with the assumption of isotropic radiation stated by the simple source model.

## Discussion

In volcano acoustics the simple source model provided by the linear acoustic theory has been commonly used to explain the pressure waves produced by volcanic activity (see ref.^27^ for a review). However, the monopole source can also be seen as an equivalent acoustic source due to a mass/volume fluid injection in an environment at rest^9^. From this perspective, the simple source has been used to infer explosive gas emission also for asymmetric acoustic signals at Mount Erebus (Antarctica)^28,29^ and, in spite of the violent explosive out-fluxes dynamics, also during Vulcanian explosions at Sakurajima volcano (Japan)^8,30^, Karymsky (Kamchatka)^16^ and Tungurahua (Ecuador)^17^ with encouraging results. However, from all these studies it is unclear whether and when the above assumptions remain still valid. No clear constraint is given by the theory, or related literature, on the required realistic evolution of the volumetric flow rate for discrete explosive events.

Numerical simulations, using the Lattice Boltzmann method, for a subsonic fluid injection (isothermal and single phase) with a Gaussian SRT predict pressure transient in agreement with the linear theory. The presence of important directivity and non linear effects are excluded in the studied low subsonic regime. The Lattice-Boltzamn modeling shows indeed how infrasound may be modeled as the result of a subsonic fluid injection using an asymmetric fluid flow profile (SRT). In particular, an asymmetric log-Normal SRT may generate an acoustic transient which is very similar to the asymmetric waveform characterizing blast waves and it can also explain pressure discontinuity in the signal, when measured not too far from the explosive source, the so-called Friedlander’s waveform. Nonetheless, the acoustic transient generated in the simulation does not involve any blast/shock wave dynamics and propagates at sonic speeds. The wavefield computed with LBM is always consistent with the one predicted by the linear theory, also when asymmetric SRT derived by the Friedlander equation is used. As a result, while an asymmetric waveform signature does not provide a sufficient condition to relate a recorded infrasonic transient to a supersonic dynamics and/or propagation effect, the use of the linear theory should be always supported by a detailed analysis of the explosive dynamics, especially when significant supersonic expansions are not clearly evident. The presence of a vertical sharp discontinuity in the compressive phase of the signal is not always recognizable and our modeling shows how it can produce waveforms very similar to the infrasonic transients generated by violent volcanic explosions or blast waves. Moreover, we noticed that the computed wavefield at Ma = 0.55 presents a directivity which is nearly absent in lower Ma simulations. We suggest that acoustic directivity may depend on the importance of compressibility effects and that for ${\rm{Ma}} > 0.3$ the wavefield may increasingly deviate from the simple source proposed by the linear theory. In this case, acoustic signals associated to explosions with exit velocity larger than ∼100 m/s could be interested by a wavefield directivity, which is not accounted by the linear theory. However, further investigations with a fully compressible numerical model are required.

In conclusion, LBM indicates that the asymmetry of the acoustic waveform is not a necessary peculiarity of supersonic dynamics, which makes difficult to understand when linear theory of sound can be used to infer eruption volumetric flow rates. More evidences need to be considered to constrain the source process in a well-known explosive dynamics context in addition to many other complexities of volcanic eruptions (e.g. ash particle loading, strong temperature gradients, crater walls). For instance, numerical simulations for Vulcanian explosive events, considering the sudden injection of a particle-steam mixture, have shown that the shape of the compressive phase of the first shock transient depends on the initial particle concentration^14^. Such a study for the smaller Strombolian explosions is also required. While the main conclusions of the present work are valid for fine ash poor Strombolian explosive activity, a reliable interpretation of infrasonic signals associated with fine-ash rich explosions also requires the development of a more comprehensive theoretical framework. For instance, eruptive gas-ash rich mixture are characterized by lower sound speed than pure gas implying that compressibility effects could start to play an important role even at lower exit velocities (higher mixture Mach Number) and possibly produce directivity in the radiated acoustic field. From this perspective, the LBM strategy proposed in this work, and its future developments, will help to better understand these processes and to quantify the uncertainties hidden in the assumptions of the theoretical models.

## Methods

### The Lattice Boltzmann method

On a macroscopic level, a fluid is regarded as continuous and can be described with partial differential equations of hydrodynamics, e.g. the Navier-Stokes equations (NS). Nevertheless, At a microscopic level, a fluid is made of individual molecules. Since a large number of particles are contained even in a small fluid volume (e.g. $\sim {10}^{19}$ per *cm*^3^ for air), directly solving the dynamics of all these particles and their interactions is almost an intractable problem. A statistical approach, where the macroscopic quantities of interest are the result of the collective behavior of a large number of interacting particles, is instead much more convenient. This approach is at the base of kinetic theory and the Lattice Boltzmann method, which deduces the dynamics of the macroscopic variables of a physical system characterizing the evolution equation of mesoscopic quantities, particle density distribution functions *f*(***ξ***, ***x***, *t*). Constraining the particle to a finite number of velocity directions ***ξ***_*i*_ with *i* = 1...*q* (the lattice), the quantity *f*_*i*_ ≡ *f*(***ξ***_*i*_, ***x***, *t*) is the probability of finding a particle at the time *t* in a volume *d****x*** located in the position ***x*** with a velocity ***ξ***_*i*_. The distribution functions *f*_*i*_ still contain more detailed information than the thermodynamic variables, and therefore the macroscopic quantities of interest result from the low-order moments only of the distribution function:

8 $$\rho =\sum _{i}{f}_{i}({\boldsymbol{r}},t)\,{\boldsymbol{u}}=\frac{1}{\rho }\sum _{i}{f}_{i}({\boldsymbol{r}},t){{\boldsymbol{\xi }}}_{i}$$

where *ρ* is the fluid density and ***u*** the velocity. An evolution equation for *f*_*i*_ is needed then to get the evolution of the macroscopic fluid variables. The most widely used equation for computational fluid dynamics is a discrete version of the Boltzmann BGK equation^31^:

9 $${f}_{i}({\boldsymbol{x}}+{{\boldsymbol{\xi }}}_{{\boldsymbol{i}}}\delta t,t+\delta t)-{f}_{i}({\boldsymbol{x}},t)=-\,\frac{1}{\tau }({f}_{i}({\boldsymbol{x}},t)-{f}_{i}^{eq}({\boldsymbol{x}},t))$$

where simply the change due to inter-particle collisions (r.h.s) is balanced by the particle transport (l.h.s). The role of the collision term (r.h.s) is in fact to leave *f*_*i*_ to tend towards an equilibrium distribution function ${f}_{i}^{eq}$ in a characteristic time *τ* which is then directly related to the fluid viscosity *ν*. Based on a multiscale analysis (Chapman-Enskog expansion), one can demonstrate that the specific form of ${f}_{i}^{eq}$ (and lattice topology) defines the physics of the fluid that can be taken into account by the LBM (e.g. isothermal NS, thermal NS or even beyond NS^32^). In this study, we use an optimized version of the standard LBM for aeroacoustic computations which is valid for isothermal fluid flow in the weakly compressible regimes (${\rm{Ma}}\sim 0.5$)^33^. In the case that pressure fluctuations are causing a significant temperature change, and hence affecting the sound speed, the model is expected to generate instabilities inducing divergence in the computational procedure. For all the presented conditions, the model was stable. Finally, the effect due to the presence of multiple phases is not considered.

## Electronic supplementary material

Supplementary Information
