# Supplementary material for: Lattice Boltzmann modeling to explain volcano acoustic source
Source: Sci Rep. 2018 Jun 22;8:9537. doi: 10.1038/s41598-018-27387-0 (PMC6015044; doi:10.1038/s41598-018-27387-0)
Supplement: Supplementary file 1 — Supplementary Information [file 41598_2018_27387_MOESM1_ESM.pdf]

Supplementary Information for "Lattice Boltzmann modeling to explain volcano acoustic source"

Federico Brogi[1,*,+], Maurizio Ripepe[2], and Costanza Bonadonna[1]

[1]Department of Earth Sciences, University of Geneva, Switzerland.
[2]Department of Earth Sciences, University of Florence, Italy
[*]fbrogi@inogs.it
[+]Now at: Istituto Nazionale di Oceanografia e di Geofisica Sperimentale, Sgonico, Italy and Istituto Nazionale di Geofisica e Vulcanologia, Sezione di Pisa, Italy.

Propagation speed

Here we report the measurements of propagation speed of the first acoustic transient generated by a Gaussian and Log-Normal SRT (fig. S1). The speed has been measured tracking the peak of the positive phase of the pressure transient and in both cases is consistent with the sound speed (± 1 m/s).

(a) (b)

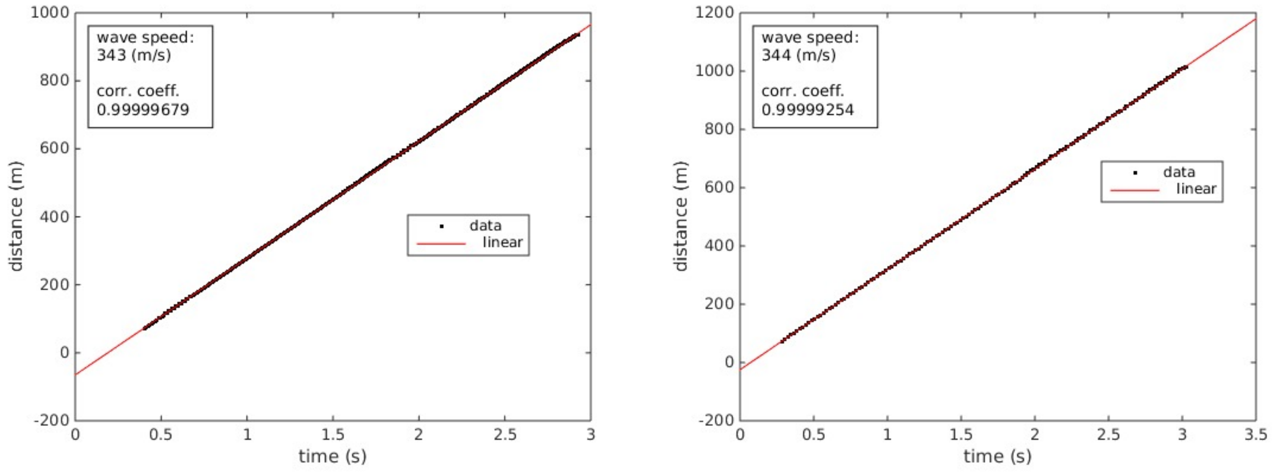

Figure S1: Sound speed measurements for the numerical simulation with (a) Gaussian SRT ($D = 10$ m, $U_{max} = 100$ m/s, $\mu_g = 1$, $\sigma_g = 0.25$, $\tau = 0.25$) and (b) Log-Normal SRT (b) ($D = 10$ m, $U_{max} = 100$ m/s, $\mu_l = 0$, $\sigma_l = 1$, $\tau = 0.25$)

.

Directivity

Here we report the directivity characterizing the computed wave fields for the of the first acoustic transient generated for different SRT and Ma (fig. S2). The directivity of the radiated wave field, and hence its degree of inconsistency with the simple source model of the linear theory, appears to be sensitive to Ma. Let us note that the LBM used for the computation may be not fully accurate on the amplitude at higher Ma. The Friedlander SRT at low Ma exhibits a slighlty higher directivity (supplementary fig. S2) but this is mainly due to the presence of weak spurious numerical artifacts along the flow direction (fig. 1e).

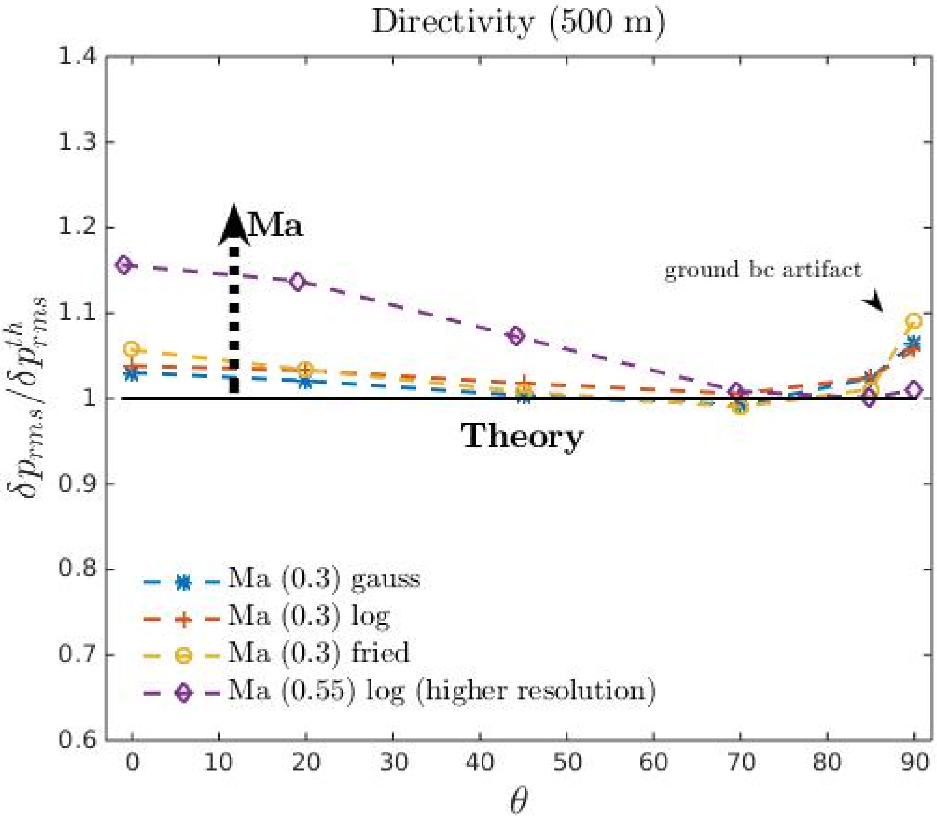

Figure S2: Directivity and deviation from the linear theory (simple source) for simulations with different SRT and Ma as measured by probes at different angles from the vertical/jet axis (90° for ground measurement, 0° for the probe on the vertical axis). For each angle θ we report the ratio $RMS(\delta p)/RMS(\delta p^{th})$, where δp is the time acoustic signal computed with LBM and δp^{th} the one predicted by the linear theory for a simple source (e.g. eq. 5).
